# Applying Reinforcement Learning to Protect Deep Neural Networks from Soft Errors

**DOI:** 10.3390/s25134196

**Published:** 2025-07-05

**Authors:** Peng Su, Yuhang Li, Zhonghai Lu, Dejiu Chen

**Affiliations:** 1 Department of Engineering Design, KTH Royal Institute of Technology, 10044 Stockholm, Sweden; pensu@kth.se (P.S.);; 2 Thrust of Microelectronics, Hong Kong University of Science and Technology (Guangzhou), Guangzhou 511458, China; zhl@hkust-gz.edu.cn

**Keywords:** reinforcement learning, soft errors protect, fault injection

## Abstract

With the advance of Artificial Intelligence, Deep Neural Networks are widely employed in various sensor-based systems to analyze operational conditions. However, due to the inherently nondeterministic and probabilistic natures of neural networks, the assurance of overall system performance could become a challenging task. In particular, soft errors could weaken the robustness of such networks and thereby threaten the system’s safety. Conventional fault-tolerant techniques by means of hardware redundancy and software correction mechanisms often involve a tricky trade-off between effectiveness and scalability in addressing the extensive design space of Deep Neural Networks. In this work, we propose a Reinforcement-Learning-based approach to protect neural networks from soft errors by addressing and identifying the vulnerable bits. The approach consists of three key steps: (1) analyzing layer-wise resiliency of Deep Neural Networks by a fault injection simulation; (2) generating layer-wise bit masks by a Reinforcement-Learning-based agent to reveal the vulnerable bits and to protect against them; and (3) synthesizing and deploying bit masks across the network with guaranteed operation efficiency by adopting transfer learning. As a case study, we select several existing neural networks to test and validate the design. The performance of the proposed approach is compared with the performance of other baseline methods, including Hamming code and the Most Significant Bits protection schemes. The results indicate that the proposed method exhibits a significant improvement. Specifically, we observe that the proposed method achieves a significant performance gain of at least 10% to 15% over on the test network. The results indicate that the proposed method dynamically and efficiently protects the vulnerable bits compared with the baseline methods.

## 1. Introduction

With the advancement of Artificial Intelligence (AI), Deep Neural Networks (DNNs) are widely employed in sensor-based systems across various fields [1]. In these systems, DNNs usually are utilized in the perception of complex operational conditions by dealing with unstructured high-dimensional data. However, the extensive parameters within the DNN lead to the computational process that is difficult to interpret and explain, posing challenges for quality management of DNNs. In particular, the ability of DNNs to withstand potential functional faults is considered to be an important factor in the safety critical systems, such as Automated Driving Systems (ADSs) [2] and medical equipment [3]. As typical functional faults, soft errors caused by radiation, aging, device variation, and temperature [4] can affect a small proportion of the data within DNNs [5]. Consequently, they weaken robustness and threaten safety by leading unexpected behaviors and system failure. Some related work [4,5,6] shows soft errors such as bit-flips that could lead to the DNN misclassifying a critical object (e.g., a truck) as a trivial one (e.g., a bird). Based on the reasoning of related system hazards, industrial standards (e.g., IEC 61508 [7] and ISO 26262 [8] ) advise systematic approaches to guarantee functional safety by fault avoidance, fault tolerance, and fault detection [9]. In addition, soft errors—particularly bit-flips—can also act as a form of attack that threatens the security of distributed intelligent systems, such as the Internet of Vehicles (IoV) and Unmanned Aerial Vehicle (UAV) swarms [10]. For example, sensor data integrated into IoV systems is often used by DNNs to determine vehicle positions. An attacker could exploit this by flipping specific bits to falsify the input data [11]. Therefore, enhancing the security of these systems requires a thorough understanding of, and protection against, the impact of soft errors on DNNs.

Conventional fault-tolerant techniques [12], such as replicating critical components via Triple Module Redundancy (TMR) [13] or inspecting vital outputs via symptom of neurons within DNN [14], pose challenges in efficiently and effectively achieving fault tolerance. Such challenges often arise from the difficulty in effectively addressing and identifying vulnerabilities within DNNs. For example, while recent studies suggest that certain layers of DNNs are more susceptible [2,15], such findings may not provide sufficient granularity for redundancy design or checksum validation in certain safety-critical applications, particularly in embedded systems with AI techniques, where physical sizes and economic costs are limiting factors [16]. Current studies propose contributions from various strategies [4,17], including optimizing DNN architectures, algorithms, microarchitecture, and hardware implementation to address these issues. These approaches enable DNN protection and reliability analysis across the functional and technical designs, as well as the Validation and Verification (V&V) process [18].

However, there remains a need for increased effort in protecting DNNs from soft errors by dynamically and efficiently identifying vulnerable bits. Meanwhile, the identification of vulnerable bits can play a critical role during functional design exploration within safety-critical systems (e.g., ADS) to support the design of necessary safety mechanisms that provide the protection [18,19,20]. Therefore, we propose a Reinforcement Learning (RL)-based method to protect DNNs from soft errors. Regarding soft errors impacting DNN performance, the RL-based agent dynamically selects and protects vulnerable bits by generating bit masks. To improve the efficiency of the proposed method, we adopt transfer learning across DNNs by utilizing the layer-wise resiliency acquired from fault injection simulation as the prior knowledge for reducing the training time. As a case study, we select different DNNs to test and validate the proposed approach. Compared to several baseline methods, the results indicate that our approach decreases the cost of redundancy and improves the protection efficiency. The contributions of this article are summarized as follows:

- Proposing a methodological framework integrating fault injection, layer-wise resiliency analysis and learning-based agent to reveal and protect DNNs against soft errors.
- Designing a layer-wise RL-based agent to identify vulnerable bits in each layer. This layer-wise learning agent efficiently and dynamically generates bit masks to protect DNNs from soft errors.
- Adopting transfer learning to improve the training efficiency and flexibility in selecting protected bits by using layer-wise resiliency as prior knowledge.

The rest of this paper is organized as follows: Section 2 presents related work on fault injections within DNNs and the protection of DNNs from soft errors. Section 3 describes the methodology and workflow of the proposed framework, which consists of fault injection, resiliency analysis, the configuration of the DRL-based agent, and the design of transfer learning. In Section 4, we use a customized MLP and ResNet-18 with MNIST and CIFAR-10 datasets to test and verify the proposed approach. The conclusion and future work are detailed in Section 5.

## 2. Related Work

In this section, we review recent studies on fault injection techniques and fault models in the context of DNNs by elaborating the adaption of fault injection techniques. Next, we present approaches aimed at improving resiliency and protecting DNNs from soft errors and discuss their limitations. To this end, we also present a background of Reinforcement Learning and its current adoption within the DNN.

### 2.1. Injecting Faults Within DNNs

Fault injection is a common approach to revealing the system-wide impacts of specific faults for requirements engineering, system and component design, and verification and validation [21]. Based on the characteristics of faults of concern, fault injection techniques for DNNs can be categorized into (1) functional fault injection, (2) technical fault injection, and (3) physical fault injection.

**Functional fault injection** involves the use of Platform-independent Models (PiMs) to simulate and analyze the functional impacts of faults [19,22]. This technique is typically applied during early design phases for revealing the sensitivity of DNN [17,23] applications regarding faults commonly represented as bit-flips, stuck-at-0/1, and Gaussian noise with certain prior distributions. The injection affects either the structure or the parameters of specific DNN models [2,5,15,23,24,25], with the scope of injection ranging from the network layers to specific neurons and parameters. By enabling an analysis of fault behaviors early in system development, functional fault injection constitutes a basis for identifying additional requirements on DNN robustness, resilience, and protection. While injecting functional faults with high fidelity into applications can often become very challenging, current studies have shown that a systematic approach to fault modeling and fault injections can still yield effectiveness similar to more detailed hardware simulation [26]. Many current approaches [2,5,15,24,25] treat the injections of faults as individual and uncorrected tasks, highlighting a significant area for improvement.

**Technical fault injection** is a technique used to assess the robustness and reliability of DNN using Platform-specific Models (PsMs) or detailed hardware-dependent solutions. It is focused on the fault behaviors of DNNs when implemented with hardware resources like CPU and AI accelerators [19,27,28,29]. The injections can be applied to the full DNN systems by targeting the microarchitectures, including Instruction Set Architectures (ISAs) and Register Transfer Level (RTL) properties, commonly through advanced virtual simulations (e.g., gem5 [30]). The aim is to effectively capture the actual system’s responses to hardware faults [26,27]. This type of injection is important for the detailed technical design of DNN systems, where the specification of hardware performance, robustness, and resilience are of particular importance.

**Physical fault injection** relies on gaining physical access or close proximity to the target DNN systems in their actual operational environments to intentionally introduce physical faults [22]. As an example of physical fault injection, prior studies [6,27] arrange the hardware (e.g., CPU and General Purpose—GPU) to be exposed to neutron beams. The results conclude that while the data corruption rate remains similar, physical fault injection exhibits a significantly higher system failure rate compared to one of the other fault injection methods. Beyond the synthetic simulations, this type of fault injection (e.g., the beam test) often requires high-cost equipment, such as radiation sources, to induce data corruption in memory. Moreover, beam tests typically offer a statistical perspective on the impact of fault models across the devices, such as Failure In Time (FIT), which does not allow faults to be measured at the level of individual memory cells or mapped to specific parameters of DNNs [31]. Therefore, this method is tailored for reliability analysis during the final technical development phase, such as with physical microchips or products.

### 2.2. Protecting DNNs from Soft Errors

Robustness has been considered as a key concept in the design of DNN protection. Previous research [4,5,6,32,33] refers to the robustness of DNNs as their capability to resist faults and maintain performance under various disturbances. The corresponding protection strategies for DNN systems can be broadly categorized as follows [33]:

**Passive strategies** are characterized by a system that works without interfering with the faults. Such approaches usually design redundant modules (e.g., triple module redundancy) to filter the faulty values by voting for the major one [34,35]. Considering the extensive parameters of DNNs, such a redundancy design could significantly increase the hardware dimensions. Some research selects critical neurons with redundant modules for fault tolerance [25,35]. However, such approaches can become inefficient due to the challenges of identifying and addressing critical neurons in large-scale DNNs. Moreover, it is normally a challenging task to protect all potential critical neurons from various errors [4,5]. Adding noise into the training data enhances the robustness of DNNs by avoiding overfitting [36]. Building on this training-based enhancement, recent studies also address the protection of DNNs against soft errors by introducing fault injections during training to improve robustness [37]. For example, a soft error library is developed to inject into the training dataset in [38]. However, these optimization-based approaches typically assume that faults occur in the input data rather than within the DNNs themselves (e.g., weights or biases of activation functions). This assumption may underestimate the impact of soft errors as DNNs inherently have the ability to filter certain unexpected inputs (e.g., values with extremely large magnitudes) through their activation functions. As an improvement, some works add regularization terms in loss functions to resist potential soft errors inside the DNNs [39]. These modifications incorporating regularization terms only provide a generic improvements of soft error resilience in DNNs. However, such a regularization may actually demand extensive iterations to converge the training process.

**Active strategies** refer to the introduction of a specific mechanism that dynamically manages resources to mitigate the impacts of faults by addressing their locations [40]. One key effort is to detect errors in the DNN architectures. Some methods employ and optimize the checksum mechanisms for detecting errors [41] in the DNN accelerator. However, such an optimization requires additional computational overhead to implement the design [42]. Other research proposes to detect errors based on data-driven approaches (e.g., machine learning techniques [43,44]). Such approaches usually need a complete comprehension of fault behaviors, requiring massive training data.

Another effort in supporting active fault tolerance is to protect computational units within DNNs. Specifically, these efforts can be categorized as follows: (1) Layer-wise protection refers to analysis of layer-wise features from DNNs. For example, a median feature selection scheme [45] is proposed to filter the impact of bit errors from each layer of the DNNs. Similarly, some work [2,46] reveals resiliency and correct errors by analyzing data distributions extracted from the intermediate layers of the DNNs. Although this approach improves the robustness by synthesizing layer-wise median features across DNN, such a fine-grained fault-tolerant module needs sophisticated data analysis techniques to extract the features from each layer, which limits its application on the large-scale DNNs. (2) Neuron-level protection refers to techniques that make DNNs recoverable from errors with dynamic constraints on the output value [24,47]. For example, an output of activation functioning larger than a predefined threshold could dramatically affect the performance of the DNNs [24]. Therefore, this work proposes a clipped function to constrain the maximum output. Although this approach mitigates the faulty behaviors by solely suppressing the output values of neurons, it still cannot provide a guarantee to correct the constrained value. (3) Bit-level protection is a fundamental solution for protecting DNNs from soft errors as it directly and explicitly safeguards the basic units within a DNN. In particular, the bit-level protection supposes to cope with Silent Data Corruption (SDC) [5], which usually refers to the corruptions in data without any explicit indication caused by flipping bits [4,17]. These bits are identified as vulnerable bits, which require protection. Several approaches [48,49] based on the Error Correction Code (ECC) are used to protect the DNN from soft errors. For example, RL-based approaches in [49] exhibit adaptability to select different modes by analyzing the power consumption and error-correcting schemes. However, these schemes are more efficient to protect bit-level data in conventional applications, such as bit streams in the communication systems. Some research studies [50,51] propose RL-based methods to address vulnerable bits in the context of DNN hardware. For example, an RL-based selective scheme [50] is proposed to protect DNNs by identifying critical bits. The agent protects the DNNs by masking the vulnerable bits across the network. Nevertheless, the extensive amount of DNN parameters could lead to the agent spending an unaffordable amount of time performing satisfactorily. To solve this issue, a compositional mechanism is used in [51] by designing global and local agents to boost the training of the RL. For instance, to protect a ResNet-18 [52] with more than 15 layers, the methods need to design local agents for each layer. Each of these local agents obtains independent local reward functions [50,51] to analyze the DNN performance. Meanwhile, a global agent with an additional reward function is used to evaluate the protection efficiency of these layer-wise agents. Such a design increases the complexity to design the reward functions and the computational costs to converge the training of the agents.

### 2.3. Adopting RL to Analyze Behaviors of DNNs

Reinforcement Learning (RL) is an interactive optimization process in which an agent makes decisions and receives feedback in the form of rewards from the environment. To ensure that an agent can make appropriate decisions in varying situations, Reinforcement Learning (RL) typically relies on a quality function (Q-function) or a value function (V-function) to quantify the impact of the agent’s decisions—such as specific states or state–action pairs—on the environment. By iteratively approximating these functions, an RL-based agent can optimize its decision-making process. As environments become increasingly complex, the evaluation of the decisions is often implemented using various neural network architectures. For example, Deep Q-Learning (DQN) employs neural networks—specifically policy and target networks—to approximate Q-values in environments with discrete and low-dimensional action spaces [53]. In contrast, the Deep Deterministic Policy Gradient (DDPG) uses actor and critic networks to optimize the agent’s actions in continuous and high-dimensional action spaces [54]. Due to their learning-enabled nature, DRL approaches are widely used for optimization-related tasks such as communication and networking in Internet of Things [55] and anomaly detection [56]. In particular, given the extensive state spaces associated with neural networks, DRL-based methods offer a promising approach for understanding and analyzing their behaviors and performance by formulating them as optimization problems. For example, DRL-based agents are widely employed to optimize network pruning by identifying and removing redundant components within a DNN. In such cases, the agent’s actions involve pruning specific parts of the target DNN—which serves as the environment to be compressed—while the states represent the DNN’s performance in response to these actions. One example is the use of a DRL-based agent for layer-wise pruning using the DDPG algorithm [57]. Building on this idea, a multi-agent design is proposed to perform channel-wise pruning within the DNN [58]. Although most existing approaches leverage DRL-based methods to optimize DNN architectures by targeting coarse-grained structures (e.g., layers or channels), only a few focus on using DRL to address vulnerabilities at a fine-grained level, such as individual parameters.

As mentioned above, this work proposes an RL-based method to protect DNNs from soft errors by addressing and identifying vulnerable bits during functional development phase. After reviewing related work on fault injection techniques, we propose a functional fault injection service featuring a simulated memory hierarchy model. With the adoption of layer-wise resiliency analysis, the DQN-based agent protects vulnerable bits from soft errors by generating bit masks within a specific layer. To enhance the efficiency of protection and reduce the time cost to deploy the agent across DNNs, we utilize resiliency analysis as prior knowledge to adopt transfer learning.

## 3. Methodology

**Figure 1:**
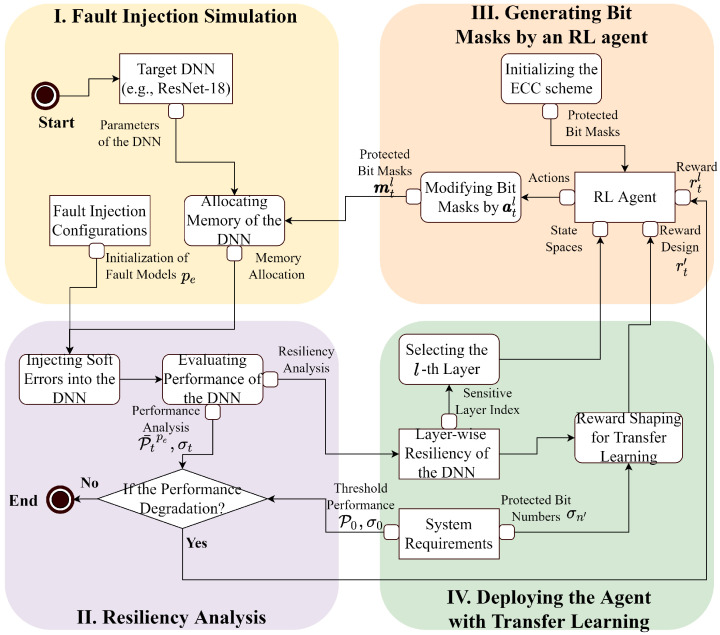
The proposed framework of applying RL to protect DNN from soft errors.

This work proposes a framework to protect DNNs with an RL-based agent that helps to reveal the vulnerable bits due to soft errors. As shown in Figure 1, we utilize a fault injection service to assess the impacts of soft errors on the DNN (Task I) and generate a layer-wise resiliency analysis (Task II). Based on the resiliency analysis in Task II, a layer-wise RL-based agent is trained to protect the vulnerable bits from soft errors by generating bit masks (Task III). To promote the training efficiency and employ the RL-based agent across DNN, we adopt transfer learning to utilize the acquired knowledge from a trained agent and resiliency analysis (Task IV).

### 3.1. Task I: Simulating Faults Within DNNs

**Figure 2:**
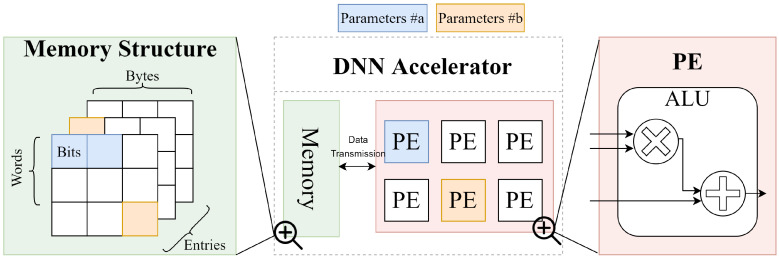
A simple structure of DNN accelerator. Depending on the DNN layer-wise characteristics (e.g., the dimensions), the parameters from the PE could be saved either in the same entry or different entries of the memory. Therefore, the fault rate $p_{e}$ could impact the parameters (e.g., parameter #a and parameter #b) when they store in different entries.

As previously mentioned, current functional fault injection methods typically manipulate parameters without considering memory resource constraints [2,5,15,24,25]. As a result, faults are usually injected into parameters as independent and identically distributed (i.i.d.) events, disregarding the occurrence of faults in the context of memory allocation. To address this issue, the proposed framework introduces an simulated memory model for fault injection within the DNN parameters. The design of this simulated model follows the memory hierarchy of DNN accelerators. Specifically, a DNN accelerator usually consists of the Processing Elements (PEs) to process the data and memory hierarchy to store the parameters of the PE [4,5]. Compared with potential faults occurred in the PE, previous research [4,5,6,32] shows that fetching data (e.g., parameters of DNNs) from the memory hierarchy could be more vulnerable due to the highly frequent operations. Therefore, we mainly focus on the fault occurring in parameters of DNNs within the memory. To inject these faults, we firstly present a schematic of a typical DNN accelerator, as shown in Figure 2.

The memory hierarchy usually organizes extensive memory cells by words and entries so that each operation fetches a batch of parameters from specific entries. To characterize the operations from different entries, we allocate the parameters including weights and bias to this memory hierarchy. Such a hierarchy is analogous to the HBM2 memory, which is a common type of Dynamic Random Access Memory (DRAM) used in DNN accelerators [6,26]. To be specific, the parameters of the target DNN are converted to a generic machine-readable format (e.g., floating bytes) and then stored in the simulated memory representing the memory hierarchy. To design this simulated model for supporting the faults to be injected, we configure the memory hierarchy, which is similar to the memory in [6]. This memory contains multiple entries to store batches of parameters as a set of continuous words. These words are strings of bytes converted from the parameters of DNNs. A batch of parameters from the same layer is stored in the same entry in sequence until all of the parameters are loaded or these entries are fully stacked.

To define soft errors occuring in these units, we assign each entry *i* with an independent error distribution $r_{e}^{i}$. Meanwhile, we use Bit Error Rate (BER) $r_{b}$ to indicate the number of bit errors per entry. To this end, the fault probability $p_{e}$ of the simulated memory for storing the parameters of DNNs can then be formulated as follows:   

(1) $$p_{e}=\sum_{i=1}^{N_{e}}r_{e}^{i}\cdot r_{b}$$

where $N_{e}$ refers to the number of entries, determined by the number of parameters to be stored in the simulated model and the size specification for each entry.

**Figure 3:**
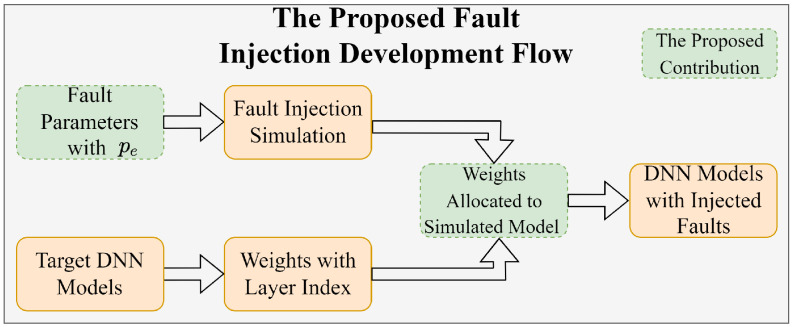
The development flow of the proposed fault injection service. The dash box represents the contribution of our proposed method.

To this end, we present the development flow based on the proposed fault injection service shown in Figure 3 as follows: (1) creating a simulated model by configuring the size of entries and words; (2) extracting the weights from pre-trained DNN models; (3) allocating the weights sequentially based on the constraints of the simulated model; (4) injecting the soft errors by assigning fault parameters within Equation (1). Specifically, the fault injection simulation is configured by injecting bit-flips in the weight values of the DNNs, with a fault probability $p_{e}$ affecting different memory entries. Additionally, compared to the most of current work which randomly inject faults across the parameters, the proposed fault injection along with the surrogate model enables the allocation of fault occurrences with respect to the memory index of the DNN’s weights.

### 3.2. Task II: Analyzing Layer-Wise Resiliency of DNNs

Once these faults are injected, we analyze the impacts on the DNNs based on a metric that measures in the context of applications. For example, the deviation of TOP-1 accuracy could be a metric that reflects the resiliency of supervised learning (e.g., image classification), while the F1 score could be used for evaluating the semi-supervised and unsupervised learning (e.g., prediction and regression). To deal with the inherent probabilistic nature of the DNNs, we analyze the resiliency by collecting a set of performance data and their deviations, which are formulated in Equation (2).

(2) $$\left\{\begin{array}{cc} {\bar{P_{t}}}^{p_{e}}=\frac{\sum_{j=1}^{N_{k}}P_{j}}{N_{k}} & \\ \sigma_{t}=\sqrt{\frac{\sum_{j=1}^{N_{k}}{\left(P_{j}-{\bar{P_{t}}}^{p_{e}}\right)}^{2}}{N_{k}}} \end{array}\right.$$

where $P_{j}$ refers to the measured performance figure of injecting faults with $p_{e}$ on the DNNs in the *j*-th execution. $N_{k}$ refers to the total number of fault injection simulation cases for collecting the performance data after injecting faults at timestamp *t*. $\sigma_{t}$ refers to the deviations caused by the injected faults.

### 3.3. Task III: Generating Bit Masks by a RL-Based Agent

A conventional error protection and correction scheme (e.g., Hamming Code) usually protects a few arbitrary bits from soft errors [59]. Although such a protection scheme has been widely used, it is insufficient for soft errors with multiple vulnerable bits. Moreover, such protection schemes usually protect arbitrary bits without considering their significance in the context of DNN operations. However, given the extensive parameters of the DNNs, efficiently addressing the vulnerable bits across the DNNs is challenging due to the following reasons: (1) Soft errors can have varying impacts across the DNNs—specifically, errors occurring in different layers can lead to different levels of performance degradation and result in varying numbers of vulnerable bits. (2) With more neurons in the layers, analyzing and addressing vulnerable bits become inefficient due to the need to inject faults across a proportion of neurons. (3) With deeper networks, it is time-consuming to identify vulnerable bits by iterating layer by layer. To address these challenges, an RL-based solution is developed to dynamically and efficiently identify the vulnerable bits by interacting the agent and the environment. The backbone of the interaction is to create a Markov Decision Process (MDP) that contains the following elements [60,61]: 〈S,A,T,r,γ〉. S refers to the state space, and A refers to a set of actions. $T(s_{t+1}|\left(s_{t},a_{t}\right))$ describes transition dynamics from the current states $s_{t}$ to the next states $s_{t+1}$ at timestamp *t* by taking an action $a_{t}$. *r* refers to an immediate reward regarding consequences of a policy π(s,a) based on the state–action pairs $\left(s_{t},a_{t}\right)$. A discount factor γ∈[0,1] is used to offset the impact of the immediate reward *r*. To evaluate the long-term rewards *R* of current state–action pairs, we use the following function [61]:

(3) $$Q^{\pi }\left(s_{t},a_{t}\right)=E\left[R|s_{t},a_{t},\pi \left(s_{t},a_{t}\right)\right]$$

**Figure 4:**
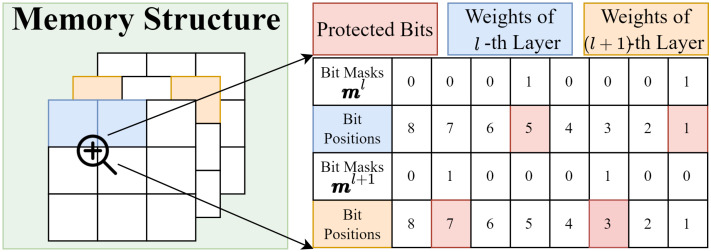
An example to illustrate that bit masks protect weights from different layers by selecting vulnerable bits.

The configuration of actions and states considers the following aspects to encourage the training of the RL agent: (1) It is impractical to implement the protection scheme with small memory granularity (e.g., each weight assigned to an individual bit mask) in the memory hierarchy. Therefore, the protection scheme should be flexible to be deployed in the context of DNNs. (2) The protection scheme should leverage the resiliency in terms of layers to decrease the protection cost (e.g., the protection number of bits). Thus, we protect the parameters (e.g., weights) by specifying a layer-wise bit mask. To be specific, we propose an RL-based agent by generating bit masks to protect specific bits in the *l*-th layer. Such a bit mask can be formulated as follows:

(4) $$m^{l}=\left(m_{0}^{l},m_{1}^{l},\ldots ,m_{w-1}^{l}\right)\in {\left(0,1\right)}^{w}$$

where *w* refers to the number of bits requiring to store a parameter from the *l*-th layer. $m_{i}^{l}=1$ refers to protect bit *i* of every weight in the layer *l*. By selecting the protected bits of parameters (e.g., weights in a layer of the DNNs), the state space S contains bit mask with different combinations, which dynamically determines the efficiency of the protection. As an example, Figure 4 shows that the weights of the DNNs store in different memory locations. By assigning the bit masks regarding the resiliency, it is efficient to protect the parameters of a layer from soft errors.

As shown in Figure 1, the actions of the RL-based agent involve marking the bit positions within the masks $m^{l}$ to explore and exploit the vulnerable bits. We define the action $a^{l}$ as follows:

(5) $$a^{l}=\left(a_{0}^{l},a_{1}^{l},\ldots ,a_{w-1}^{l}\right)$$

where $a_{i}^{l}\in \left\{0,1\right\}$ refers to the actions for configuring the bit masks to protect bit position *i* at layer *l*. The primary reason for selecting binary representations of actions is to reduce the size of the state and action spaces. The size of the action space using this binary representation is |A|, denoted as $2^{w}$, which is less than the agent sample’s actions from a continuous distribution (e.g., a Gaussian distribution).As an example of generating bit masks via current actions, Figure 4 presents how the initial bit masks $m_{0}^{l}=\left(0\right)$ are modified by actions $a_{t}^{l}$ at step *t*, where $a_{1}^{l}$ and $a_{5}^{l}$ are set to 1 to produce $m_{t}^{l}$. In addition, to encourage the agent to explore and exploit vulnerable bits in the next step, it receives rewards based on the policy $\pi (m_{t}^{l},a_{t}^{l})$.

We design the reward function by considering the following aspects: (1) The reward should encourage the generation of bit masks by the RL-based agent to protect the parameters of DNNs from soft errors. (2) The reward should balance the performance and redundancy cost by exploring and exploiting vulnerable bits. Therefore, the reward function designed for layer *l* at timestamp *t* is modeled as follows:

(6) $$r_{t}^{l}=\alpha \cdot f_{i}\left(P_{0}-{\bar{P_{t}}}^{p_{e}}\right)-\beta \cdot f_{p}\left(\sum_{i=0}^{w}I\left(m_{i}^{l}\right)\right)$$

where $P_{0}$ denotes the performance of the DNNs defined by the system requirements. ${\bar{P_{t}}}^{p_{e}}$ derived from Equation (2) refers to the performance after injecting faults with the fault probability $p_{e}$. The functions $f_{i}$ and $f_{p}$ are scaling functions that normalize the performance and protected number of bits to facilitate the convergence of the DRL-based agent. Specifically, $f_{i}\left(\cdot \right)$ is a function where the agent receives a greater reward when there is less discrepancy between the $P_{0}$ and ${\bar{P_{t}}}^{p_{e}}$. $f_{p}\left(\cdot \right)$ represents that when more bit needs to be protected, the agent receives more penalty. When a specific bit is masked by $m_{i}^{l}$, the indication function $I(m_{i}^{l})=1$. α,β refer to the coefficients to balance the performance and the number of masked bits.

Based on the modeling of the design space and reward functions of the RL-agent, we conclude the states and actions are discrete with numerical rewards. Therefore, we adopt Deep Q-learning (DQN) [53], a model-free and off-policy algorithm, to approximate the optimal action-value functions $Q^{*}\left(s,a\right)$, which is represented as follows:

(7) $$Q^{*}\left(s,a\right)=max_{\pi }E\left[R_{t}|s_{t}=s,a_{t}=a,\pi \left(s,a\right)\right]$$

where a refers to the action for marking bits defined in Equation (5), and s refers to the bit masks defined in Equation (4).

To stable optimize and approximate the Q-value, the DQN utilizes the similar implementation in [53], where this Q-network contains a target network and a policy network with the same configurations and parameters. The weights of the target network are slowly updated by the learned policy network. We model this training process as Equation (8), which minimize loss function $L_{i}\left(\theta_{i}\right)$ at each iteration *i*:

(8) $$L_{i}\left(\theta_{i}\right)=E_{s,a∼\rho (\cdot )}\left[{\left(r_{i}^{l}+\gamma \cdot Q\left(s,a;\theta_{i-1}^{\prime }\right)-Q\left(s,a;\theta_{i}\right)\right)}^{2}\right]$$

where ρ(·) refers to the probability distribution of the state–action pairs. $\theta_{i-1}^{\prime },\theta_{i}$ refer to the parameters updating for the target and policy networks. γ refers to the discount factor for the long-term reward. To encourage the agent to explore and exploit using off-policy approaches, the DQN implements an ϵ-greedy policy to select and execute actions by sampling from the replay memory, which is a buffer pool over many episodes during training. The replay buffer supports breaking the sequential correlations that arise during online training by using past experiences stored in the buffer. In addition, by adopting the replay memory, the agent allows for greater data efficiency by updating the weights from the experience within the replay memory. Moreover, by sampling episodes from the replay memory, correlations within the sample states are broken, increasing the training efficiency of the agent.

### 3.4. Task IV: Deploying the Agent with Transfer Learning

Although this DRL-based agent could protect the parameters in a specific layer, such a protection needs to become more effective across the DNNs. Considering the complexity of the state and action spaces, it is impractical to train such an agent for each layer in terms of time consumption and scalability. To illustrate these issues, we utilize a five-layer neural network as an example: the action space of bit masks contains $2^{w}$ actions to protect weights within each layer; to obtain all potential optimal actions simultaneously following the methods in [50,51], the action space increases from $2^{w}$ to $2^{5w}$. In addition, with an increase in the number of neurons in deeper layers, training the layer-wise DRL agent becomes more time-consuming due to the faults’ need to be injected across a proportion of neurons. To cope with this issue, transfer learning provides a solution to accelerate the learning process by reusing the knowledge of a trained agent with similar scenarios [62]. Additionally, with the results from the fault injection service, the layer-wise resiliency of the DNNs can expose prior knowledge for designing the reward functions. Such prior knowledge could stimulate the convergence of training the agent [63]. As a common method of transfer learning, we adopt the reward shaping by formulating the MDP as follows [62,64,65]:

(9) $$M=<S,A,T,r,\gamma >\to M^{\prime }=<S,A,T,r^{\prime },\gamma >$$

where M and $M^{\prime }$ refer to the source and target domain for transferring the knowledge. To be specific, M is the MDP, which is trained at the *l*-th layer in Section 3.3. $M^{\prime }$ is the MDP for the other layers. $r^{\prime }$ refers to the newly shaped rewards, denoting to $r^{\prime }=r^{l}+r_{e}$. We specify $r_{e}$ as $r_{e}^{n^{\prime }}$ regarding the layer index $n^{\prime }$, where $n^{\prime }\neq l$. We define the reshaped reward $r_{e}^{n^{\prime }}$ as Equation (10) for balancing the number of protected bits with the prior knowledge concluded from the fault injection service.

(10) $$r_{e}^{n^{\prime }}=-\tau \cdot \left(\sum_{i=0}^{w}I\left(m_{i}^{n^{\prime }}\right)-\sigma_{n^{\prime }}\right)$$

where τ refers to a coefficient to adjust the importance of the prior knowledge for training agents. $m_{i}^{n^{\prime }}$ refers to the protected bit *i* in a specific layer $n^{\prime }$. The number of protected bits estimated by the DQN-based agent is formulated as $\sum_{i=0}^{w}I\left(m_{i}^{n^{\prime }}\right)$. By combining the knowledge from bit masks trained in the specific layers with the resiliency analysis, $\sigma_{n^{\prime }}$ refers to an approximated number of protected bits. For example, $\sigma_{n^{\prime }}$ could be larger in some non-trivial layers (e.g., the first layer of the DNNs) than others. This threshold is determined by the resiliency of each layer under fault injection, which helps reduce the search cost within the state space. When the number of protected bits estimated by the DQN-based agent exceeds the threshold defined by prior knowledge, the agent receives a penalty and is encouraged to continue exploring. Conversely, if the estimated number of protected bits is within the threshold, the agent receives a reward and is encouraged to exploit its current policy to optimize performance. Equation (10) implies that transfer learning encourages the agent to protect the layers regarding approximated protected bit numbers from prior knowledge. In particular, when attaining a similar performance, the agent receives a penalty if the amount of bit masks from the agent is more significant than the approximated one.

## 4. Case Study

We follow a similar experimental design to that of existing works [57,58] by selecting an MLP and a ResNet-18 and evaluating them on the MNIST and CIFAR-10 datasets to test and validate our framework. We implement the case study in the following steps: (1) Identifying the layer-wise resiliency of these networks by simulating the fault injection. (2) Conducting the training process of the RL-based agent for a specific layer. (3) Measuring the time consumption of the agent training with other layers by transferring the knowledge. (4) Evaluating the performance of bit masks generated by the proposed method.

### 4.1. Evaluating Resiliency by Injecting Faults

**Figure 5:**
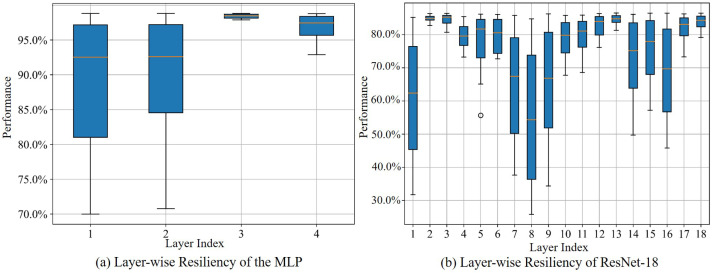
Layer-wise resiliency of DNN by injecting faults with 500 iterations.

Considering the energy efficiency and occupation of bit storage for large-scale neural networks, more and more hardware platforms intend to utilize 8-bit fixed points (INT-8) instead of 32-bit floating points to convert and store the parameters of the DNNs [66]. However, a side effect by using the INT-8 is that the DNNs require more precision and reliability to maintain the performance [67]. To meet these requirements, we test and validate our design by converting the weights of the DNNs to INT-8 and storing them in the proposed simulated model mentioned in Section 3.1. Given the significance and severity of soft errors [4,5], including bit-flips and stuck-at-0/1 errors, we inject bit-flips—recognized as the most critical faults affecting DNN performance—into the weights of the simulated model according to Equation (1). The model is configured with an entry size of 256 bits, with each size storing four 64-bit words. With different parameters, we collect the metrics to evaluate the performance and robustness of the DNNs by using Equation (2). Specifically, we present the layer-wise resiliency evaluated under $r_{b}=10\%$, and $r_{e}^{i}$ follows the normal distribution with $\sum_{i=1}^{N_{e}}r_{e}^{i}=1$ in Figure 5. The number of $N_{e}$ depends on the number of weights within the target DNN model, and they are automatically sequentially allocated with an entry size of 256 bits. Figure 5a illustrates that Layer 1 and 2 could dramatically affect the performance when soft errors occur. Additionally, Layer 3 shows a better resiliency compared to the others.

Based on the resiliency of this MLP, we generalize that the amount of protected bits is correlated to the layer index. For example, the neurons in the intermediate layers could require less protected bits than those in other layers. However, such a correlation is more complex when the layers become deeper in ResNet-18. Layer 1 of the ResNet-18 shows a similar pattern as the MLP. Meanwhile, Layers 7, 8, and 9, allocated in the middle of the network, are more sensitive to soft errors. Such a phenomenon shows that the various DNN architectures could differ regarding the performance of a unified protection scheme (e.g., conventional ECC scheme), indicating the demand of a tailored scheme regarding the DNNs.

### 4.2. Training the DRL-Based Agent with a Specific Layer

From Figure 5, we observe that input and intermediate layers are significantly affected by the soft errors. Considering the extensive parameters in the intermediate layers (e.g., Layer 8 of the ResNet-18 contains more than 140,000 parameters), we select Layer 2 of the MLP and Layer 1 of the ResNet-18, which have fewer parameters compared to the intermediate layers, to serve as the environment for the DRL-based agent. The agent consists of two MLP networks as a target network and a policy network. Each of them contains three hidden dense layers with sizes of 64, 128, and 128, and the output neurons of the first two hidden layers are activated by Leaky Relu functions. The generated actions from the last layer is activated by a Relu function. We configure the initialized bit masks as $m_{0}=\left(0\right)$. The discount factor is set as γ=0.99 with the learning rate $l_{r}=0.001$. During the training of the agent, we use the Adam optimizer for stochastic gradient descent. We adopt the ϵ-greedy algorithm to balance the efficiency of exploring and exploiting with different state–actions pairs. At the start of the training episodes *M*, ϵ could be configured with 0.5. After some iterations of the training episodes, we improve the efficiency to exploit the optimal actions by increasing the value of ϵ.

We propose the requirements with $P_{0}$ and $\sigma_{0}$ to indicate the threshold of the desired metrics of the DNNs. To converge the training of the DRL-based agent, we mark the terminal conditions for ending the loop of each episode as the bit masks satisfy the requirements instead of iterating the entire loop. Such an implementation could decrease the amount of training episodes and avoid the over-fitting. The overall training algorithm incorporated to the fault injection service is listed in Algorithm 1. When the performance and deviation in different episodes always meet the requirements (line 14 in Algorithm 1), then the agent is treated as converge.
**Algorithm 1** Training the DRL-based Agent for a Specific Layer**Require:** Configurations of the agent                                                            ▹ such as $\gamma ,l_{r},l$**Ensure:** Tailored bit masks $m^{l}$ for a specific layer *l* 1:**for** k←1 to *M* **do**                                                                        ▹ Start episodes, M=200 2:Initialize the bit masks $m_{0}^{l}$ 3:Configure the fault parameters $p_{e}$                                                               ▹ Equation (1) 4:Inject faults into a specific layer *l* 5:      **for** t=1 to *T* **do**                                                                     ▹ Start iterations, T=500 6:            Run a random seed $p_{t}$ 7:            **if** $p_{t}\geq p_{\epsilon }$ **then**                                                                           ▹ϵ-greedy algorithm 8:                  Select a random action $a_{t}^{l}$ 9:            **else**10:                  Select an action $a_{t}$ by the DQN                                    ▹Equations (6) and (7)11:            **end if**12:            Execute $m_{t}^{l}\leftarrow a_{t}^{l}$                                                                   ▹ Equations (4) and (5)13:            Protect the weights with bit masks $m_{t}^{l}$14:            **if** $\bar{P_{t}^{p_{e}}}\geq P_{0}$ and $\sigma_{t}\leq \sigma_{0}$ **then**                                                             ▹ Equation (2)15:                  Break the iteration and start new episodes16:            **else**17:                  Continue the iterations18:            **end if**19:            Optimize DQN by gradient descent                                                ▹ Equation (8)20:      **end for**21:**end for**22:Execute $m^{l}=m_{t}^{l}$23:**return** $m^{l}$

**Figure 6:**
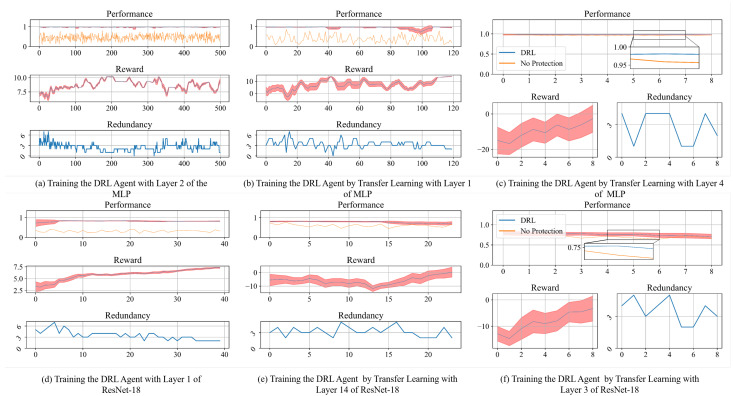
Snapshot during training the agents with their performance, rewards, and redundancy. The red boundaries within the figures denote the upper and lower values observed across various training episodes.

A sample of training the agent with the MLP and ResNet-18 can be found in Figure 6a,d. The upper and lower bounds of the red region in the performance’s curves refer to ${\bar{P}}_{t}\pm \sigma_{t}$. All these tests run on the hardware configuration with Intel i7-9750H and NVIDIA Geforce GTX 1660 Ti. In these experiments, we configure $r_{e}^{i}$ as a normal distribution and $r_{b}=20\%$. We set $f_{i},f_{p}$ as a scaling function with α=1.15 and β=1 to normalize the output. By converging the training of the agent, we observe that the bit masks finally stabilize to maintain the performance by protecting a few bits of each weight. To be specific, the optimal bit mask of the MLP is $m_{mlp}^{2}=\left(1,0,0,1,0,1,0,0\right)$, and the one of the ResNet-18 is $m_{res}^{1}=\left(1,1,0,0,0,0,0,0\right)$. Additionally, we find that the agent deployed in ResNet-18 requires around 100 episodes (27,675 s) to converge, spending much less time compared with the one of the MLP (31,668.79 s). The explicit reasons is that the number of weights could affect the time consumption of training the agent. To be specific, parameters of Layer 1 in the ResNet-18 (9472) are less than the ones of Layer 2 (12,800) in the MLP. Another potential reason could be that with deeper layers of the ResNet-18, it has a better optimization of the network topology compared with the MLP designed by ours.

### 4.3. Ablation Study of Transfer Learning

In Section 4.2, we find that training a layer-wise agent in MLP and ResNet-18 requires 31,668 s and 27,675 s. Obviously, this time cost is impractical to learn all the layers with the similar workload. In addition, with more neurons in the deeper layers, the training of the agents is more time-consuming. Therefore, regarding Equations (9) and (10), we use transfer learning by modifying the rewards and reusing the agent in Algorithm 1. We configure τ=0.5 to leverage the current and approximated bit masks. According to the bit masks $m_{mlp}^{2},m_{res}^{1}$ learned from Section 4.2, we specify $\sigma_{n^{\prime }}=2$ for the rest of the layers of the MLP. Considering the complexity of the ResNet-18, $\sigma_{n^{\prime }}=3$ is used for the critical layers, which are those that exhibit significant performance degradation by fault injection simulations in Figure 5 (e.g., Layers 7, 8, and 9). $\sigma_{n^{\prime }}=2$ is used for the rest of the parts of the networks (e.g., Layers 2, 3, and 4). To implement transfer learning in the remaining layers, we reload the parameters of the DRL-based agent trained by Algorithm 1 after the training concludes, as described in Algorithm 1. Next, the DRL-based agent with transfer learning continues the training with reshaped rewards $\sigma_{n^{\prime }}$ following Equation (10). The terminal states of this agent follow the condition defined in line 14–15 in Algorithm 1. In Figure 6b,e, we present the performance of bit masks by adopting transfer learning in Layer 1 and Layer 14 of the MLP and ResNet-18, which are sensitive layers to soft errors. Compared with Figure 6a,d, these agents spend fewer episodes to converge the training while still maintaining the performance of the DNNs. Moreover, these layers’ protected bits are approaching even less than the bit numbers $\sigma_{n^{\prime }}$ defined by prior knowledge. Figure 6c,f refer to adopting the transfer learning to train the agents for the robust layers shown in resiliency analysis. The results from these layers, including Layer 4 of the MLP and the Layer 3 of ResNet-18, present that the agents use a few episodes to attain acceptable bit masks. Although soft errors in these layers could slightly impact the performance, the bit masks still show superior results to the baseline.

**Figure 7:**
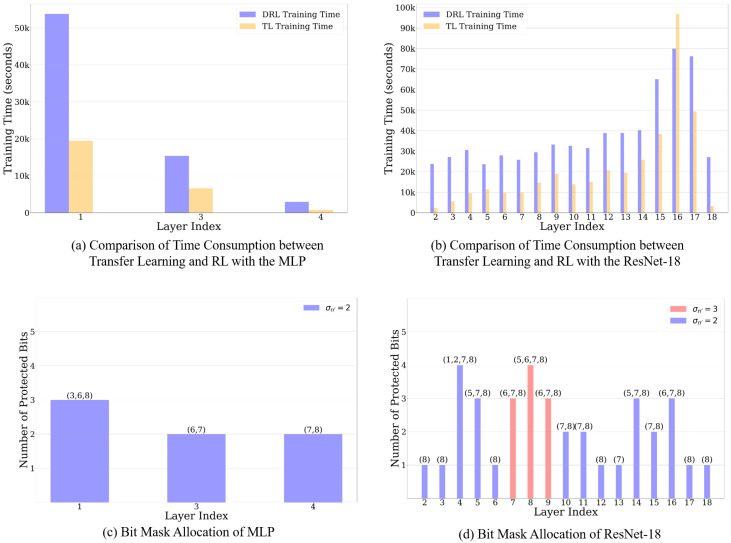
Overall results by adopting transfer learning in different layers. The protected bit position is shown in the top of the bar.

To this end, we conduct an ablation study in Figure 7 by comparing the adaptation of transfer learning with the training approach outlined in [50,51], where the layer-wise agents are trained to protect bits across the entire DNN simultaneously. In this figure, we also present the number and index of vulnerable bits by configuring different $\sigma_{n^{\prime }}$ during transfer learning. Figure 7a,b present the comparison of time cost between using RL and transfer learning. By adopting transfer learning, the trend of training time consumption is significantly decreased. Compared to the RL-based method without transfer learning, the training time of the agent for the MLP is reduced by an average of 62.76%, decreasing from 72,170 s to 26,874 s, and for ResNet-16, it is reduced by 44.03%, decreasing from 652,225 s to 363,991 s. We observe that there is one exception in Figure 7b, where Layer 16 of ResNet-18 spends more time than the RL-based training method. The potential rationale behind this phenomenon lies in balancing the resiliency of the layers with the $\sigma_{n^{\prime }}$ assigned by prior knowledge. Such a design attempts to spend more time to reach the desired performance as well as the optimal number of protected bits. In fact, the agent in this layer suggests that the bit masks protect 3 bits, while $\sigma_{n^{\prime }}=2$. Such a conflict implies the consistency of the rationale. Figure 7c,d show the number of protected bits and their allocation in each layer. The labels of each bar refer to the positions protected by bit masks. Although we find that the agents protect the significant bits of the weights, these agents still discover other bit positions that affect the performance. In addition, $\sigma_{n^{\prime }}$ with different values determines the number of protected bits to a certain extent, decreasing the redundancy cost to protect the DNNs. For example, half of the layers defined $\sigma_{n^{\prime }}=2$ select one bit to protect the weights in Figure 7d.

### 4.4. Ablation Study of the Proposed Method

The bits highlighted by the bit masks could be protected by different practical schemes such as the BCH code and Hamming code. We utilize a similar comparative analysis as in [50,51] by selecting the Hamming Code for the ECC scheme to protect the bits within the weights. We use the MSB method and conventional Hamming code, which protects significant and arbitrary bits from soft errors, as a benchmark to compare with the proposed method. To further analyze the protection efficiency of the proposed framework, we also design a comparative scheme to protect *k* bits (referred to Top-k), where faults most frequently occur. The number of protected positions, *k*, is the same as those selected by the DRL-based agents shown in Figure 4c,d. We primarily assess the proportion between information and redundant bits to present the effectiveness of these methods. Next, we introduce a SDC ratio to underscore the protection efficiency. Finally, we measure the performance of the DNNs under various soft error rates by adopting different protection schemes. All these evaluations are based on the following configurations: $r_{e}^{i}$ follows the normal distribution, $r_{b}$, with 10% and 20% to reveal that the SDC, which could be visible, affected the performance of the DNNs.

We evaluate the efficiency of the protection schemes by modifying the metrics from [50,51], where they measure the proportion between the information and redundant bits.

(11) $$\eta =\frac{k_{pro}\cdot \left(n_{i}-k_{i}\right)}{k_{i}}$$

where $\left(n_{i},k_{i}\right)$ refer to a linear code with $n_{i}$ bits and $k_{i}$ information bits for a weight in the DNNs; $k_{pro}$ refers to the protected bits in the information bits. Equation (11) indicates the redundancy rate to protect the same bits in a weight by using different schemes. With the same measurement in [50,51], the average redundancy rate for the proposed method $\eta_{mlp}=50.05\%$, and $\eta_{resnet}=51.25\%$, while $\eta_{msb}=56.24\%$, and $\eta_{hamming}=75.00\%$. Since the Top-k scheme protects the same number of bits as the proposed methods, the average redundancy rate is the same as $\eta_{mlp},\eta_{resnet}$.

Due to the inherent robustness and probabilistic nature of the DNNs, comparing bit-by-bit could be invalid to reflect the degradation of the overall performance [4,5,6]. We propose a metric defined in [5,44] as Equation (12) to further highlight and evaluate the protection schemes by normalizing the SDC ratio under the specific fault probability $p_{e}$. A smaller SDC ratio indicates that a more robust DNN resists soft errors.

(12) $$p\left(SDC|p_{e}\right)=\frac{\frac{\sum_{i=1}^{N_{t}}\left(P_{g}-P_{i}\right)}{N_{t}}}{P_{g}}=\frac{P_{g}-{\bar{P}}_{N_{t}}^{p_{e}}}{P_{g}}$$

where $P_{g}$ refers to the performance of the DNNs when they are error-free (golden run). $N_{t}$ refers to the times of running DNNs with different protection schemes under the fault injection. We configure $N_{t}=500$ across these experiments. ${\bar{P}}_{N_{t}}^{p_{e}}$ is the same as Equation (2), referring to the average performance of the DNNs protected by different methods in $N_{t}$ tests.

In Table 1, we list the SDC ratio which is computed by Equation (12). Although we observe that the Hamming code scheme outperforms other baseline solutions, it suffers a higher average redundancy rate than other baseline methods. On the other hand, the MSB and Top-k schemes demonstrate varying protection efficiency across different layers and DNN structures. Specifically, we observe that the performance of the MSB scheme tends to be better than that of the Top-k scheme in less sensitive layers of ResNet-18. This is because Top-k protects only a few bits, the same number as the DRL-based agent shown in Figure 7, which is fewer than the number protected by the MSB scheme. Beyond these baseline methods, the proposed method significantly decreases the SDC ratio under different BERs, improving the robustness of the DNNs. Compared to baseline methods that only protect a few crucial or random bits, we conclude that this improvement stems from the reward design of the RL-based agent, which effectively explores vulnerable bits within weights. In particular, the proposed method shows better performance than the Top-k scheme, which protects the same number of bits, indicating a higher protection efficiency of the proposed method. For MLP or ResNet-18 at $r_{b}=20\%$, the average performance of the proposed method decreases from 98.9% to 97.67% and from 89.60% to 80.80% when protecting the critical layers, including Layers 1 and 2 of the MLP and Layers 1, 7, 8, and 9 of the ResNet-18. These results still significantly outperform those of the baseline schemes. Meanwhile, the SDC ratios significantly decrease to 1.24 within MLP and to 7.54 within ResNet-18. In addition, our method performs superior results in ResNet-18, implying that it is more suitable for neural networks with deeper layers.

**Figure 8:**
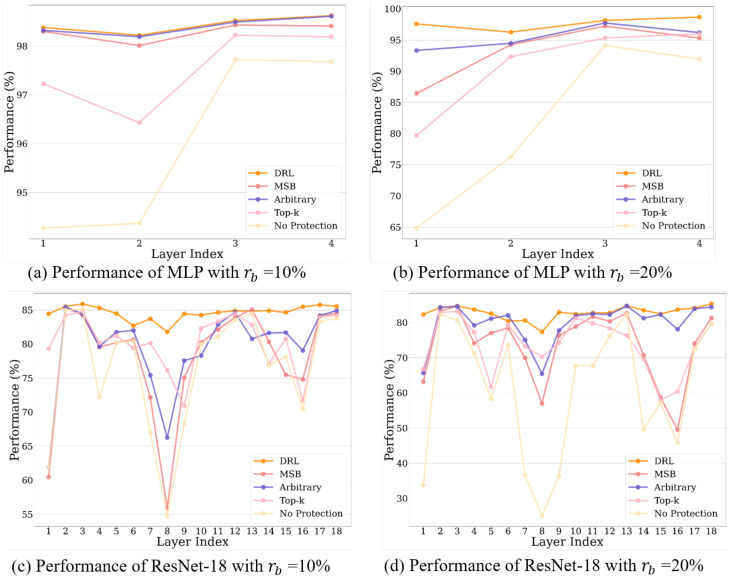
Performance between different protection schemes with soft errors.

To further evaluate the propose methods, we illustrate the overall performance of the DNNs using both the proposed method and the baseline methods across the varying soft error rates in Figure 8. Although these methods demonstrate similar performance in the MLP with $r_{b}=10\%$, as depicted in Figure 8a, the proposed method outperforms them as $r_{b}$ increases. In particular, the proposed methods demonstrate significant improvement compared to the baseline methods across ResNet-18 in Figure 8c,d, suggesting that the proposed framework is more efficient and effective as the DNNs adopt a more complex model.

## 5. Conclusions and Future Work

This paper presents a dynamic error protection method by combining RL with transfer learning to protect DNNs from soft errors. The proposed method can dynamically and flexibly identify and address the vulnerable bits from each layer of the DNNs. By comparing with the current work, we summarize the contribution of the proposed methods as follows: (1) Compared to current work in functional fault injection [2,5,15,24,25], we introduce a simulated model for injecting faults within the weights of the DNNs, which enables RL-based methods in addressing vulnerable bits in the early function design phase. (2) Compared with conventional protection schemes that protect arbitrary bits, we train a layer-wise agent with the consideration of the resiliency revealed by the fault injection service. As a result, this agent dynamically selects vulnerable bits by balancing the performance and redundancy. (3) Compared with the related work by using RL-based agents in [50,51], our work optimizes the time consumption by training a layer-wise agent instead of using global and local ones. By specifying an explicit reward mechanism, the proposed layer-wise agent intends to suppress the hyper-parameters’ impact on the agent, avoiding configuring sophisticated reward functions in [50,51]. (4) With the help of the prior knowledge acquired by layer-wise resiliency analysis, the proposed method accelerates the agent’s convergence by adopting transfer learning. The proposed method performs flexibility to configure the number of protected bits regarding the prior knowledge. Moreover, the adoption of transfer learning enhances the potential to generalize the DRL-based agent to other DNN structures by refining the reshaped rewards. (5) In the experiments, ResNet-18, which is more complex than a simple MLP, still demonstrates significant efficiency, suggesting the potential for our framework to be extended to larger and more complex DNNs.

Based on the above conclusions, we prepare to extend our work from the following aspects: (1) The implementation of protection schemes using bit masks generated by the DRL-based agent could be further strengthened by integrating it with specific hardware configurations to optimize the proposed framework. (2) The design of DRL and transfer learning can be further enhanced through various optimization techniques (e.g., knowledge distillation) to improve the training efficiency. For example, the hyperparameters within this framework can be fine-tuned in accordance with specific system requirements. Specifically, the correlation between the specification of numerical rewards and injected faults can be further parameterized to mitigate the coupling effects caused by the random fault injection presented in Task I. In addition, the algorithmic optimization can be further used in the framework to improve the training efficiency. For example, prioritized replay buffers offer an enhanced version of the standard replay buffer by sampling transitions that are less frequent or more informative, thereby improving the agent’s performance. (3) Given the detailed and realistic evaluation from microarchitecture fault injection, combining the RL-based agent with a technical fault injection seems promising. Utilizing the simulator gem5 and its fault injector gemifi, we prepare to conduct a detailed evaluation of the RL-based agent, taking into account timing and resource constraints. (4) The proposed framework can be further integrated into the design of safety-critical systems to ensure functional safety. For example, the DRL-based agent can be deployed online or offline in the learning-enabled components of the ADS to improve reliability. Specifically, during the technical design for deploying these components in hardware (e.g., FPGA/ASIC), the proposed framework provides solutions to enhance functional safety by identifying and addressing vulnerabilities in the context of bit-level data. (5) Despite the evaluation on MLP and ResNet-18 with the INT-8 quantization scheme, further experiments are needed to analyze the generalizability and scalability of the proposed framework. Specifically, we plan to implement the proposed framework in other more complex DNN structures with different quantization schemes to extract the critical layers and protect them from soft errors.

## Figures and Tables

**Table 1:** Performance evaluation between different protection schemes. The golden run performances $P_{g}$ of MLP and ResNet-18 are 98.90% and 89.6%.

DNN Type	$r_{b}$ (%)	Average Performance Protecting in Critical Layers (%)					SDC Ratio Across the DNN				
		Our Method	Without Protection	Hamming Code	MSB	Top-k	Our Method	Without Protection	Hamming Code	MSB	Top-k
MLP	10	**98.30**	94.32	98.26	98.16	96.83	**0.47**	2.92	0.50	0.62	1.40
	20	**97.67**	70.55	93.91	90.35	86.61	**1.24**	17.30	3.50	5.64	8.15
ResNet-18	10	**83.60**	62.98	70.29	65.89	76.63	**5.55**	14.63	11.17	12.93	9.91
	20	**80.80**	32.91	70.98	66.61	71.11	**7.57**	31.99	10.92	18.03	9.93

## Data Availability

Dataset available on request from the authors.
